# Colocutaneous fistula associated with Crohn’s disease following Kugel patch repair for inguinal hernia: a case report

**DOI:** 10.1093/jscr/rjac005

**Published:** 2022-02-02

**Authors:** Hiroki Sakai, Koji Kubota, Takahide Yokoyama, Akira Shimizu, Tsuyoshi Notake, Hitoshi Masuo, Takahiro Yoshizawa, Kiyotaka Hosoda, Hikaru Hayashi, Koya Yasukawa, Kentaro Umemura, Atsushi Kamachi, Takamune Goto, Hidenori Tomida, Shiori Yamazaki, Yuji Soejima

**Affiliations:** Department of Surgery, Division of Gastroenterological, Hepato-Biliary-Pancreatic, Transplantation and Pediatric Surgery, Shinshu University School of Medicine, Matsumoto, Japan; Department of Surgery, Division of Gastroenterological, Hepato-Biliary-Pancreatic, Transplantation and Pediatric Surgery, Shinshu University School of Medicine, Matsumoto, Japan; Department of Surgery, Shinshu Ueda Medical Center, Ueda, Japan; Department of Surgery, Division of Gastroenterological, Hepato-Biliary-Pancreatic, Transplantation and Pediatric Surgery, Shinshu University School of Medicine, Matsumoto, Japan; Department of Surgery, Division of Gastroenterological, Hepato-Biliary-Pancreatic, Transplantation and Pediatric Surgery, Shinshu University School of Medicine, Matsumoto, Japan; Department of Surgery, Division of Gastroenterological, Hepato-Biliary-Pancreatic, Transplantation and Pediatric Surgery, Shinshu University School of Medicine, Matsumoto, Japan; Department of Surgery, Division of Gastroenterological, Hepato-Biliary-Pancreatic, Transplantation and Pediatric Surgery, Shinshu University School of Medicine, Matsumoto, Japan; Department of Surgery, Division of Gastroenterological, Hepato-Biliary-Pancreatic, Transplantation and Pediatric Surgery, Shinshu University School of Medicine, Matsumoto, Japan; Department of Surgery, Division of Gastroenterological, Hepato-Biliary-Pancreatic, Transplantation and Pediatric Surgery, Shinshu University School of Medicine, Matsumoto, Japan; Department of Surgery, Division of Gastroenterological, Hepato-Biliary-Pancreatic, Transplantation and Pediatric Surgery, Shinshu University School of Medicine, Matsumoto, Japan; Department of Surgery, Division of Gastroenterological, Hepato-Biliary-Pancreatic, Transplantation and Pediatric Surgery, Shinshu University School of Medicine, Matsumoto, Japan; Department of Surgery, Division of Gastroenterological, Hepato-Biliary-Pancreatic, Transplantation and Pediatric Surgery, Shinshu University School of Medicine, Matsumoto, Japan; Department of Surgery, Division of Gastroenterological, Hepato-Biliary-Pancreatic, Transplantation and Pediatric Surgery, Shinshu University School of Medicine, Matsumoto, Japan; Department of Surgery, Division of Gastroenterological, Hepato-Biliary-Pancreatic, Transplantation and Pediatric Surgery, Shinshu University School of Medicine, Matsumoto, Japan; Department of Surgery, Division of Gastroenterological, Hepato-Biliary-Pancreatic, Transplantation and Pediatric Surgery, Shinshu University School of Medicine, Matsumoto, Japan; Department of Surgery, Division of Gastroenterological, Hepato-Biliary-Pancreatic, Transplantation and Pediatric Surgery, Shinshu University School of Medicine, Matsumoto, Japan

## Abstract

Colocutaneous fistula associated with Crohn’s disease after mesh repair for inguinal hernia has not been previously reported in the literature. We report such case in an 83-year-old man following a preperitoneal repair of a left-sided inguinal hernia using Kugel patch. The patient has Crohn’s disease in remission status for 4 years. One month after inguinal hernia repair, he presented with fever and left-sided inguinal pain and swelling. Computed tomography revealed abscess formation in the preperitoneal and subcutaneous space of the left-sided inguinal region. Colonoscopy showed local exacerbation of Crohn’s disease in the sigmoid colon, and formation of fistula between the sigmoid colon and abdominal wall of the left-sided inguinal region. We performed mesh removal with Hartmann resection following percutaneous abscess drainage. The post-operative course was uneventful, and no sign of recurrence of the hernia was found for 3 years post-operatively.

## INTRODUCTION

Tension-free inguinal hernioplasty using a monofilament polypropylene mesh is currently the most popular procedure. However, the implantation of a synthetic prosthesis may lead to severe infectious complications in some rare cases. We present the first case of colocutaneous fistula associated with Crohn’s disease after inguinal hernia repair using Kugel patch.

## CASE REPORT

**Figure 1:**
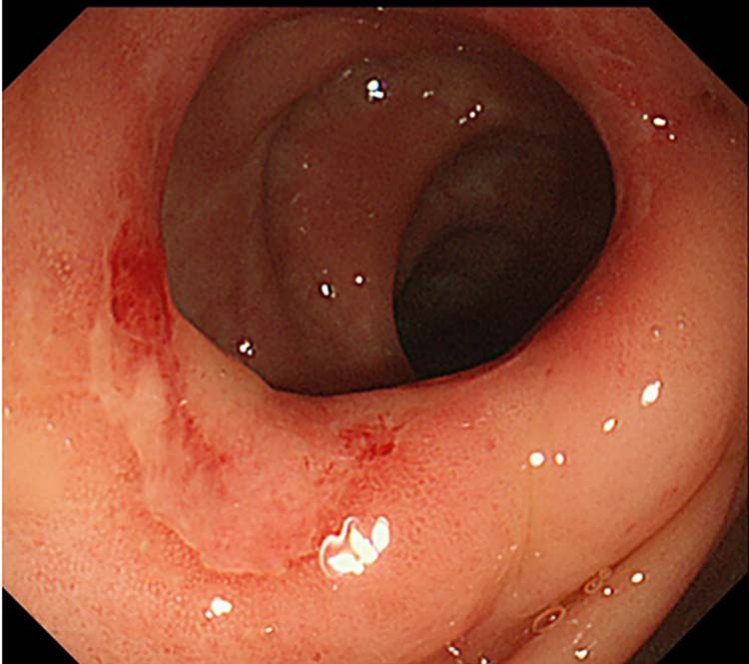
Preoperative colonoscopy findings; there was no evidence of active Crohn’s disease, such as ulceration, although erosions were noted.

An 83-year-old man was admitted to our hospital for the treatment of a left-sided indirect inguinal hernia. He had been receiving infliximab for Crohn’s disease and maintained in remission status for 4 years. A colonoscopy prior to hernia repair revealed mild inflammation in the colon without active findings, such as ulceration, stenosis or fistula (Fig. 1).

**Figure 2:**
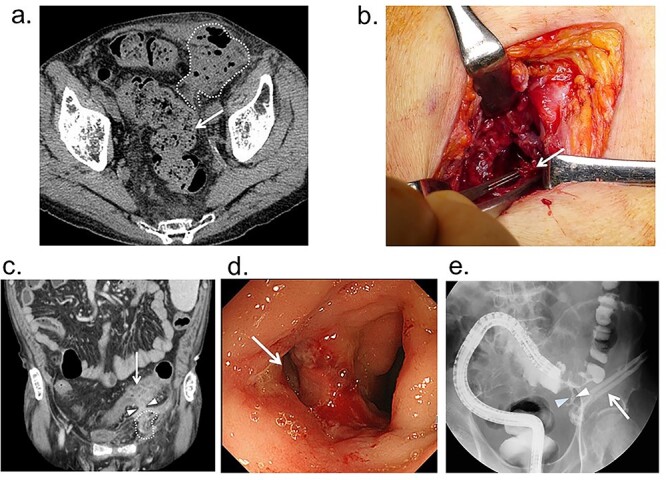
(**a**) Before drainage abdominal CT findings; abscess is found in the subcutaneous space and preperitoneal cavity of the left inguinal region (dotted-line circle), and it is adjacent to the sigmoid colon (arrow); (**b**) findings of emergency drainage; a portion of the Kugel patch (arrow) was firmly adherent to the abdominal wall, and the abscess cavity continued further into the mesh, but it was unclear whether it had reached the abdominal cavity; (**c**) CT findings after drainage: edematous changes in the sigmoid colon and a fistula continuous with the abscess cavity; (**d**) colonoscopy: ulceration and fistula (arrow) are found in the sigmoid colon; (**e**) lower gastrointestinal tract radiography findings after drainage: fistula is found (arrowhead) between the sigmoid colon and drainage tube (arrow).

He received Kugel patch repair and was discharged a day after surgery without any complication. However, he presented with fever, swelling and pain in the left inguinal lesion 5 weeks after hernioplasty and was readmitted to our hospital on the 44th post-operative day. Physical examination revealed a painful mass and inflammation on the operative scar of the left inguinal hernia. Laboratory examination showed white blood cell count of 7390/mm^3^ and C-reactive protein level of 13.2 mg/dL. Abdominal computed tomography (CT) revealed abscess formation spreading from the subcutaneous space to preperitoneal cavity of the left inguinal region, which was adjacent to the sigmoid colon (Fig. 2a). The patient underwent emergency exploration and abscess drainage through the left inguinal incision scar. Kugel patch was tightly adhered to the abdominal wall (Fig. 2b). Abscess was observed under the aponeurosis of the external abdominal oblique muscle and around the mesh. The pus from the abscess cavity grew *Bacillus subtilis*.

**Table 1:** Reported cases of colocutaneous fistula after open tension-free inguinal hernia repair

**Author**	**Year**	**Age**	**Sex**	**Type of inguinal hernia**	**Primary operation**	**Periods from primary operation to occurring fistula**	**Re-operation**	**Causes of fistula**
Zubaidi	2006	75	male	N/A	mesh plug	3 years	mesh removal and direct suture	vasculitis, steroid use, diverticular disease
Ishiguro	2009	34	male	N/A	mesh plug	3 years	partial sigmoidectomy, mesh removal, iliopubic tract repair	mesh migration into the sigmoid colon
Zuvela	2012	60	male	direct and indirect	PHS	6 years	mesh removal, left hemicastration, bipolar ileostomy, Mcvay herniorrhaphy	contact between the mesh and sigmoid colon
Al-Subaie	2015	52	male	direct	Lichtenstein	3 years	sigmoidectomy, resection of the abdominal wall with fistula tract, placing GORE®︎ Bio-A®︎ Tissue Reinforcement	mesh migration into the sigmoid colon
Sekiguchi	2015	57	male	N/A	mesh plug	13 years	mesh removal, partial cecectomy, fistula resection, direct suture	mesh migration into the cecum
Scarinigi	2016	80	male	N/A	mesh plug	26 years	laparoscopic-assisted sigmoidectomy, fistula resection and direct suture	mesh migration into the sigmoid colon
Isaia	2016	69	male	indirect	mesh plug	9 years	sigmoidectomy, mesh removal and direct suture	mesh migration into the sigmoid colon
Our case		83	male	indirect	Kugel	35 days	sigmoidectomy, colostomy, mesh removal with the abdominal wall and direct suture	relapse of Crohn’s disease

Post-operatively, he was treated with antibiotics intravenously according to sensitivity and underwent further examination to identify the cause of abscess formation. Enhanced abdominal CT performed 3 weeks after drainage revealed a cord-like structure between the abscess cavity and sigmoid colon (Fig. 2c). A colonoscopy with water-soluble contrast enema revealed local exacerbation of Crohn’s disease with ulceration in the sigmoid colon and fistula formation between the sigmoid colon to the drainage tube (Fig. 2d and e).

**Figure 3:**
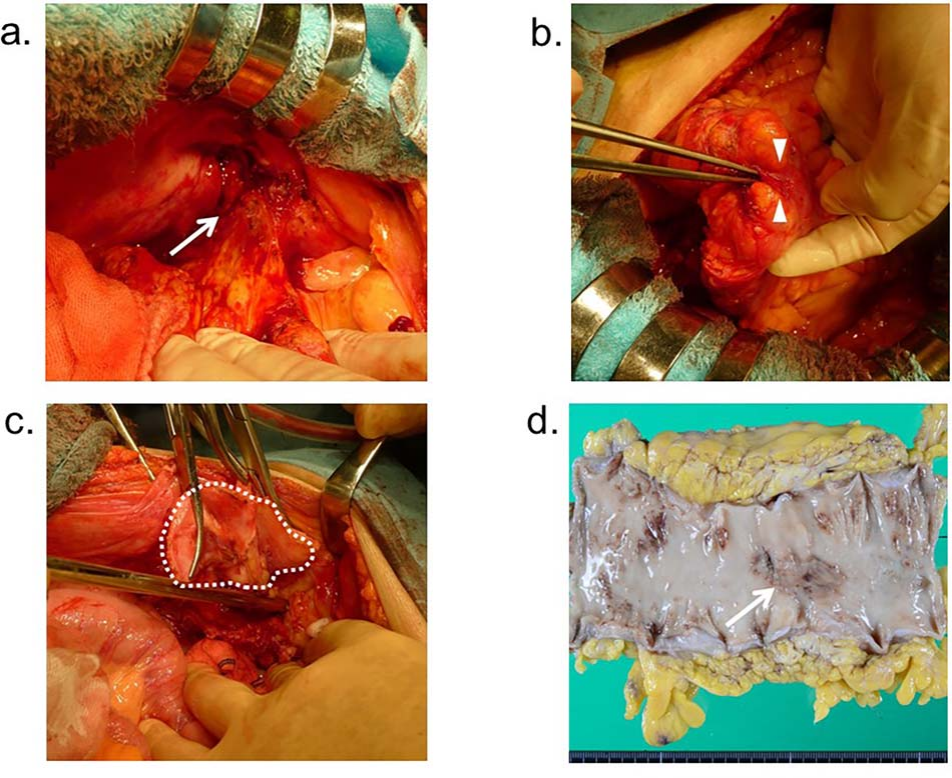
Operative findings; (**a**) the sigmoid colon adheres to the abdominal wall (arrow); (**b**) fistulous canal is observed between the sigmoid colon and Kugel mesh (arrowhead); (**c**) the mesh adheres severely to the peritoneum and posterior wall of the inguinal canal (dotted-line circle); (**d**) macroscopic findings; colonic ulcer was found in the resected specimen, and a fistula was found in the ulcer (arrow).

Based on these findings, a diagnosis of colocutaneous fistula associated with Crohn’s disease was made, and the patient underwent mesh removal with Hartmann’s procedure and fistula excision electively under general anesthesia 36 days after drainage. During laparotomy, the sigmoid colon densely adhered to the abdominal wall of the left inguinal region (Fig. 3a). After dissecting the sigmoid colon from the inguinal region, a fistulous canal was observed between the sigmoid colon and mesh (Fig. 3b). The mesh severely adhered to the peritoneum and posterior wall of the inguinal canal (Fig. 3c). Neither migration nor exposure of the mesh into the peritoneal cavity was observed. The sigmoid colon, including the fistula canal, was resected, and sigmoid colostomy was performed. The mesh was removed with the peritoneum of the left inguinal region, and the defect of the peritoneum was reconstructed and closed by direct suturing. Macroscopically, a colonic ulcer was found in the resected specimen, and a fistula was found in the ulcer (Fig. 3d).

**Figure 4:**
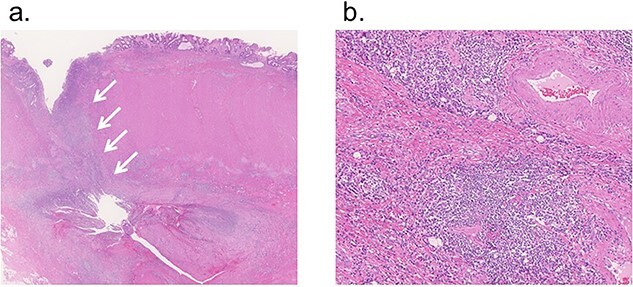
Microscopic findings; (**a**) fissuring ulcer was found in line with the fistula (12.5×, H&E, arrow); (**b**) transmural inflammation was detected in the colon wall surrounding the ulcer (100×, H&E).

Microscopically, fissuring ulcer was found in line with the fistula (Fig. 4a), and transmural inflammation was detected surrounding the ulcer (Fig. 4b). These findings were characteristics of Crohn’s disease. The patient recovered uneventfully and was discharged 2 weeks after re-operation. At 3 years post-operatively, recurrence of the left inguinal hernia was not observed.

## DISCUSSION

This is the first report on colocutaneous fistula following Kugel patch repair in a patient with Crohn’s disease. Tension-free mesh repair is definitely the global gold standard for inguinal hernia treatment. In this report, a patient with Crohn’s disease in remission underwent Kugel patch repair and re-operation due to enterocutaneous fistula formation. We report the precautions for inguinal hernia treatment in patients with inflammatory bowel disease (IBD), including Crohn’s disease.

Tomas *et al.* reported that incisional hernia (IH) repair with insertion of synthetic mesh into the inlay was associated with a 10% incidence of enterocutaneous fistulae in patients with IBD [1]. Furthermore, they reported that the incidence of late enterocutaneous fistulae was ~20 times higher than in patients without IBD. Inlay synthetic mesh for IH repair in patients with IBD increases the risk of enterocutaneous fistulas and is recommended to be avoided. Similarly, Kugel patch repair with synthetic mesh inserted in the inlay layer could be complicated by enterocutaneous fistulas. However, colocutaneous fistula after open tension-free hernioplasty for inguinal hernia is a rare complication, and there have been only eight reports including this case [2–8] (Table 1). The mesh plug technique was used in many cases [2, 3, 6–8], and the cause of colocutaneous fistulas has been reported to be mesh migration [3, 5–8] or direct contact between the mesh and colon [4]. Both causes can occur naturally due to the shape of the mesh plug. Furthermore, diverticulitis was reported as a cause of colonic fistulae [2]; however, there were no reports describing the relationship between IBD and colonic fistulae. Enterocutaneous fistula occurred 3–7 years after operation in the reported cases of IH with IBD. Reported cases of enterocutaneous fistula after inguinal hernia repair also occurred between 3 and 26 years post-operatively. Alternatively, in our case, the symptoms appeared on the 35th post-operative day; hence, the onset mechanisms might be different from other cases.

Colonoscopy performed before radical hernia repair did not show findings suggestive of inflammation in the sigmoid colon; however, colocutaneous fistula occurred after primary operation. Microscopically, fissuring ulcer was found in line with the fistula. This ulcer was a characteristic of Crohn’s disease. Moreover, mesh migration, direct contact to the sigmoid colon or diverticulitis was not found in this case. Therefore, we speculate that Crohn’s disease may have flared up and caused the Kugel patch and colocutaneous fistula.

When inserting a synthetic mesh such as Kugel patch repair or mesh plug procedure in patients with inguinal hernia complicated by IBD, it is necessary to carefully select a technique, such as the Lichtenstein method, in consideration of the layers to be inserted.
